# Unravelling travellers’ route choice behaviour at full-scale urban network by focusing on representative OD pairs in computer experiments

**DOI:** 10.1371/journal.pone.0225069

**Published:** 2019-11-12

**Authors:** Humberto González Ramírez, Ludovic Leclercq, Nicolas Chiabaut, Cécile Becarie, Jean Krug

**Affiliations:** Univ. Lyon, Univ. Gustave Eiffel, IFSTTAR, ENTPE, LICIT, Lyon, France; Monash University, AUSTRALIA

## Abstract

In a city-scale network, trips are made in thousands of origin-destination (OD) pairs connected by multiple routes, resulting in a large number of alternatives with diverse characteristics that influence the route choice behaviour of the travellers. As a consequence, to accurately predict user choices at full network scale, a route choice model should be scalable to suit all possible configurations that may be encountered. In this article, a new methodology to obtain such a model is proposed. The main idea is to use clustering analysis to obtain a small set of representative OD pairs and routes that can be investigated in detail through computer route choice experiments to collect observations on travellers behaviour. The results are then scaled-up to all other OD pairs in the network. It was found that 9 OD pair configurations are sufficient to represent the network of Lyon, France, composed of 96,096 OD pairs and 559,423 routes. The observations, collected over these nine representative OD pair configurations, were used to estimate three mixed logit models. The predictive accuracy of the three models was tested against the predictive accuracy of the same models (with the same specification), but estimated over randomly selected OD pair configurations. The obtained results show that the models estimated with the representative OD pairs are superior in predictive accuracy, thus suggesting the scaling-up to the entire network of the choices of the participants over the representative OD pair configurations, and validating the methodology in this study.

## Introduction

Urban congestion occurs when traffic demand locally exceeds the network capacity. The local demand is the combination of the global travel demand between the different origins and destinations and the travellers’ route choices, which define how many trips are made at the same place in a given time window. Thus, at a city-scale level, i.e., considering all the OD pairs and links in the network, route choice is a key determinant of urban transportation network performance. Route choice behaviour has been extensively studied in the transportation literature from two main but different angles. The first, related to human factors and mainly founded in discrete choice models [1–3], is focused on the identification of the determinants of travellers’ individual choices. This line of research is based on investigating travellers’ behaviour through experiments that consist in either observing their choices in the field (revealed preference) or asking them what would be their choices in hypothetical scenarios (stated preference). The second line of research, tackles the problem at full-scale and aims to solve the network loading problem to determine static or dynamic traffic states over all the network links. In this case, the interactions between the demand and the route choices on all the OD pairs in the network are considered altogether to define general principles that determine the network equilibrium. This is, for example, the case of the deterministic network equilibrium principle [4], that states that the travellers are selfish optimisers who only try to minimise their travel costs when choosing a route amongst all the alternatives; at the equilibrium, all the used routes that connect an OD pair have the same minimal cost.

Theoretically, the study of route choice at an individual and network level are consistent, however, in practice, there is a lack of connection between the two [5]. The reason is that, on the one hand, studies of route choice behaviour are focused in specific determinants of travellers’ route choice and, therefore, are based on simple scenarios (two or three routes in few OD pair configurations) that do not cover the multiplicity of situations that are found in a city-scale transportation network. In these experiments, particular attention has been paid to the study of how travellers learn from experience [6–8], the impact of advanced travel information systems (ATIS) [9–15], and the effect of travel time variability and risk attitudes in the travellers choices [16–18]. On the other hand, in the network loading problem, representations have been designed as a simplified mathematical abstractions that permit to calculate the network loading under different behavioural principles, such as the deterministic user equilibrium [4], stochastic user equilibrium [19] or bounded rational user equilibrium [20]. These representations often assume that the only variable influencing travellers’ route choice behaviour is the travel time, ignoring other local factors, related to the network OD configuration, that have been recognised to influence route choice behaviour [21–25]. One of the main reasons for the gap between research in individual route choice behaviour and network loading is the lack of observations at large scale over a sufficient number of OD configurations, that would allow discrete choice models to scale-up at the network level and thus enable the design of network equilibrium founded in a more user-oriented approach. The ambition of this study is to fulfil this gap by the selection of OD pairs that are representative of the OD configurations that are found in a transportation network, and then use these OD pairs in computer experiments to collect data on travellers’ route choice behaviour.

In a city-scale network, trips are made in thousands of OD pairs connected by several routes (in the case concerning this study, the city of Lyon in France, the network has 96,096 OD pairs and 559,423 routes), resulting in a large number of diverse routes, consequence of the topology of the network. For example, the route alternatives connecting an OD pair located in the central part of a city are likely to have short length, a high number of intersections and turns, but are unlikely to include segments of freeways. In contrast, the routes connecting an OD pair that traverses the city are longer and are more likely to include routes with fewer number of intersections and segments of freeways. From the point of view of the design of experiments, this implies that the number of scenarios must be reduced to a small but representative set of scenarios, such that the choices of travellers in any scenario found in the network can be approximated by a choice model estimated with this small set. More specifically, a representative set of OD pairs and routes is such that, for any randomly sampled OD pair in the network it is possible to find an OD pair in the representative set with similar attributes. Thus, assuming that the choices of travellers are similar for similar situations, an estimated model on the representative OD pairs could adequately reproduce the choices in the rest of the OD pairs. The question addressed in this work is: how to find a set of OD pairs and routes, such that it is representative of the OD configurations and route attributes found in the network, while being small enough so that a sufficient number of observations on route choices can be collected through computer route choice experiments?

The solution proposed in this work is based on *k*-means clustering [26] of the full set of OD pairs and routes in the network. In cluster analysis, the observations, in this case OD pairs and routes, are grouped in clusters characterised for having elements that are similar among themselves, but dissimilar to the elements in the other clusters. In the problem pertaining this article, the elements in a cluster will show similar orientation, length, % of freeway, directness and number of turns, and thus a cluster *C*_*i*_ will be, for example, composed mainly of OD pairs of short length in the central part of the city, with direct routes and low % of freeway composition, whereas another cluster *C*_*j*_ will be composed of OD pairs representing long trips traversing the city, with some non-direct routes composed mainly of freeways. Assuming that there are *k* clusters, the elements of a cluster can be regarded as belonging to a same class of OD pair configurations, and the whole network as being composed of elements of *k* different classes. Therefore, the OD pairs and routes in the network can be represented by elements in the *k* clusters. A natural choice to represent the elements in a cluster is the mean element in the cluster (cluster centroid), as it is the point with minimum euclidean distance to all the elements in the cluster. Thus, the cluster centroids are chosen as representative of the clusters’ elements, and the *k* clusters’ centroids as representative of the OD pairs and routes in the whole network. These OD pairs and routes are then used in computer experiments to collect data on travellers route choice behaviour. Note that the set representative OD pairs and routes found with *k*-means is representative of the attributes of the network, so the question that arises here is if a model estimated over this representative set can adequately reproduce the choices in the rest of the OD pairs in the network. To answer this question, three discrete choice models are estimated with the observations over the representative set. The discrete choice model used in this work is a joint mixed logit model (MXL), which under certain conditions, as is the case in this study, is equivalent to to the panel data formulation of MXL models [2, 27–29]. The predictive accuracy of these models are compared against the predictive accuracy of the same models, but estimated with randomly chosen sets of OD pair configurations in a sort of cross validation procedure.

The results of the above methodology are that the models estimated with the observations over the representative OD pair configurations are better in predicting the route choices on *unseen* OD pairs, i.e., on OD pairs not used for the estimation process. On the one hand, these results demonstrate how a careful selection of OD pairs for experiments on route choice behaviour can improve the results of a choice model in a broader set of OD pairs and, on the other hand, that cluster analysis can be used to find these OD pair configurations. These findings have direct implications for urban traffic simulators, which solve the network loading problem to determine the time-evolving traffic states in the network. The scalable route choice model proposed in this paper can be implemented in such simulators without adding significantly computational complexity, compared to the usual simple equilibrium rule, e.g., user equilibrium. Furthermore, the use of clustering techniques to find the most relevant OD pairs and routes in the network, provides an efficient method to calibrate route choice models that can be easily replicated in any urban transportation network.

## Materials and methods

### Participants in the route choice experiments

The data on route choice behaviour in this article comes from six route choice experiments carried out between February 2018 and February 2019. The participants in the experiments were students at the University of Lyon taking part in the courses of traffic theory (66%), staff from the IFSTTAR (French Institute of Science and Technology for Transport, Development and Networks) and other universities, who received an invitation by e-mail to remotely join the experiments via a web browser (34%). All participants have signed, before the experiments begin, an informed consent form describing the objectives of the study, the data collection and processing, and the confidentially rules. Participants could opt out of the experiment at any time. No personal data were mandatory to participate to the experiments as people had the opportunity to identify themselves by a login of their choice. Finally, all data were fully anonymised and processed as such. At the beginning of the experiments, the participants were briefed about the objective of the experiment and the interface of the experimental platform; for the participants that joined the experiments via web, a document with the instructions was shared. The participants were instructed to choose the route that they consider the *best* to complete a trip on time.

Three of the six experiments were specifically implemented for the purpose of this work, so they were configured to obtain observations in the 9 representative OD pairs. The rest of the experiments were implemented for previous studies, so they were configured over 21 OD pairs different from the 9 representative set; data coming from these experiments was used to validate the methodology in this work. Throughout the six experiments, 3,334 choices of 483 participants were recorded, from which 802 choices of 73 participants were made over the nine representative OD pairs. In the experiments, the participants were confronted to several route choice problems in the different OD pairs, the task of the participants was to choose one of the three alternative routes to complete the trip before a given time.

### Obtaining representative OD pairs and routes

#### Data: The Lyon network

**Fig 1:**
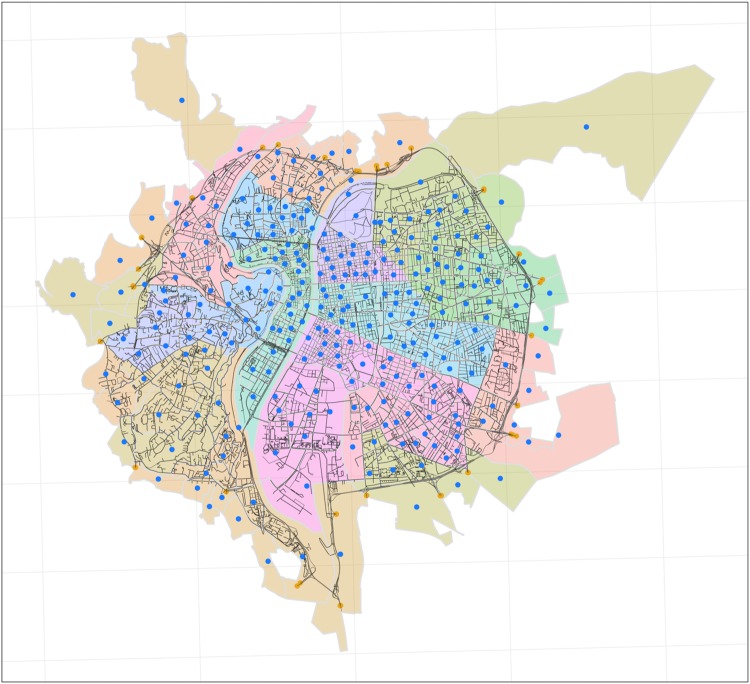
Lyon road network. The zones are depicted in different colours with their centroids in blue. The entry/exit points to the network are depicted with yellow points. There is an average of 5.82 routes connecting each origin and destination. The geodata used to render the plot is from ©OpenStreetMap contributors, licensed under the Open Data Commons Open Database License (ODbL). The zones are from Contours…Iris^®^ licensed under the Open Licence v1.0 from Etalab. The maps were rendered using The R Project for Statistical Computing which is distributed as Free Software under the terms of the Free Software Foundation’s GNU General Public License.

The origins and destinations in the network of the city of Lyon, France, come from the zoning by the National Institute of Statistics and Economic Studies (INSEE) [30], and the major entry/exit points to the network. The zones are the geostatistic units used for the trip demand estimations and represent the origins and destinations of the trips generated or terminated inside the network. The entry and exit points represent the origins and destinations of the demand coming or going outside the network. In total, there are 285 zones, 29 entry points and 28 exit points in the Lyon network. The total number of origins is 313 and of destinations 310 (this quantity does not correspond exactly to the sum of zones plus entries/exits as there are zones that may have no outgoing or incoming trips), giving a total number of 96,096 OD pairs (see Fig 1). The most likely routes joining the origins to the destinations are derived with the A* algorithm looking for the *k*-shortest paths in free-flow travel time. This is roughly equivalent to minimising the travel distance, but accounting for the influence of spacial limits. The total number of routes in the network, obtained with this algorithm, is 559,423, with an average number of 5.82 routes per OD pair.

#### Data representation

The selected route features in this study are the informed travel time, the length, directness, number of turns per kilometre and the percentage of freeway in the route composition. This features were selected as they are variables relevant in travellers’ route choice behaviour [21–25], and because they are the attributes that participants can observe in the computer route choice experiments. In the experiments, the number of routes connecting each OD pair is limited to three. This limitation, however, does not restrict the scope of the experiments or diminishes the quality of the results for two reasons. First, choice sets with many alternatives may be burdensome for participants as they may have trouble identifying the differences between the routes. Second, the low variability between routes attributes due to the small number of alternatives in the choice set is compensated by the presence of many OD pairs, that are considered by jointly estimating a random utility model.

**Fig 2:**
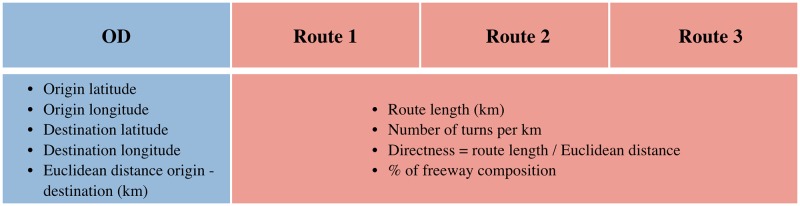
OD-routes vector. The vector is composed of the attributes of the OD pair and the three routes connecting the origin and destination, with *length*(*Route*_1) ≤ *length*(*Route*_2) ≤ *length*(*Route*_3).

An OD pair and three routes connecting the origin and destination, defined as *OD-routes*, are characterised by the variables describing the origin and the destination (latitude, longitude and the euclidean distance between them), and the variables describing the three routes connecting them (the length of the route, the number of turns per kilometre, the directness of the route, and the percentage of freeway in the route). An OD-routes is then defined by 17 variables: 5 OD pair specific and 12 describing the routes (4 for each route). An OD-routes is represented as a vector in which the attributes of the three routes appear ordered by length, from shortest to longest. A depiction of the OD-routes objects is shown in Fig 2.

For the clustering of the OD pairs and routes, and thus the route choice experiments, the short routes (less than 1.5 km) and highly overlapping routes belonging to the same OD pair (sharing more than 70% of their links) were not considered. The reason is that very short trips lack of real alternatives: usually there is an unique route to travel from origin to destination. The highly overlapping routes are removed from the analysis because, from a route choice experiment perspective, the similarity between the routes may cause participants not to consider some routes as real alternatives and, furthermore, highly overlapping routes lack of the variability required for a choice model to capture the impact of each route attribute in the choices. After removal of the very short trips and the high overlapping routes, the OD-routes are obtained by considering all the possible combinations of three routes from the set of routes joining that particular OD pair. For example, if there are 5 routes joining an OD pair, then the total number of OD-routes that are obtained is (53)=10. The total number of OD-routes in the network is 624,490.

#### Clustering of the OD-routes

Before clustering, the data was normalised so that all the variables describing the OD-routes have the same weight in determining the dissimilarity between observations; this step is necessary when the range of the variables are not comparable, as is the case in the OD-routes where the directness of the routes takes values in the interval (0, 1), but the length of the routes takes values in the interval (0, 35). The OD-routes are clustered using *k*-means with euclidean distance, determining the optimal number of clusters, k*, using the *elbow method* [26]. The idea behind the elbow method is to select the optimal number of clusters k*, such that the mean dissimilarity of the elements in the clusters does not decrease significantly with the k* + 1 clustering. The measure of dissimilarity of the elements in a cluster is the within-cluster sum of squares (WCSS), i.e., the sum of the square distance between the elements in a cluster. One of the OD-routes among the 1% nearest to the theoretical cluster centroid is selected as the cluster centroid. This is done because the theoretical centroid, i.e., the mean of the variables of the elements in the clusters, may not be part of the data.

### Route choice experiments

The route choice experiments were carried out using a computer platform, the mobility decision game (MDG), that has been developed in the LICIT laboratory to investigate travellers’ decisions in transportation networks at large scale. The network description in the MDG is based on the full map of a real road network: the city of Lyon in France in our experiment. To generate the scenarios in which the choices are made, the MDG interacts with a single dynamic microscopic simulator, based on the LWR traffic model [31], which generates and handles all the trips that populate the network. To produce the simulated scenarios, the microscopic traffic simulator takes as an input the OD pairs in the network, their corresponding trip demands and the most important alternatives connecting the origins to the destinations. In the MDG, the participants access simultaneously to the experiment through a web interface, consisting of the map of the city of Lyon, France, road network. Each participant receives periodically new specific *missions*, that consist in travelling from an origin to a destination by choosing one of the three alternative routes that are proposed. The choices of the participants are considered as updated trip specifications by the microscopic simulator that runs in a central server. This alters the traffic conditions in the network. During a MDG session, multiple OD pairs are assigned to the participants, allowing to observe the choices of the same participants in different OD pairs. Furthermore, some of the participants receive traffic information as travel time estimates for the different route options in a given mission, allowing to assess the impact of travel time information in the decisions of travellers. Thus, the MDG permits to investigate the determinants of the participants’ decisions under different conditions (traffic conditions, traffic information and route characteristics).

### Route choice model

Random utility models (RUMs) have been broadly used to understand and predict the route choice of travellers [7, 11, 12, 17, 18]. Joint RUMs arise in situations in which decisions of the same individuals are observed in several related choice problems, and correlation among their decisions is suspected. This is the case of surveys, where the answers of individuals to different questions may be correlated; or in route choice, with decisions of travellers in different OD pairs. A special case in joint RUMs is when the choice problems share part of their variables. In this situation, the coefficients of the shared variables in the model can be assumed to be equal across the choice problems. This problem is encountered when combining different data sources, as in [32], where the authors developed the techniques to jointly estimate a multinomial logit model (MNL) combining reveal preference and stated preference data to study the switching of mode of transportation of travellers. Other related works can be found in [33–37]. When the choice problems share all of their variables, then the joint RUMs consists of an unique representation of the utility, given by the variables and their respective coefficients, which is equivalent to a panel data RUM. This is the case here, as several models are estimated, one for each OD pair. However, as the OD pairs are described by the same variables, the coefficients can be assumed to be equal for all the OD pairs. Therefore, the utility of the joint model is reduced to a single representation, and the model can be estimated as a panel data model.

The joint model for route choice, used in this study, is based on the mixed multinomial logit model (MXL) for panel data [2, 27–29]. The MXL is a generalisation of the MNL in which the coefficients are assumed to be random, accounting for the heterogeneity in individuals’ preferences. Furthermore, estimation of MXLs are easily extended to account for observations of repeated choices by the same individuals, i.e., panel data. Formally, when decisions of individuals are observed in several choice situations, the utility that an individual *i* gets from alternative *j* in the MXL model is written as

$$\begin{array}{c} U_{ijs}=x_{ijs}^{T}\beta_{i}+\varepsilon_{ijs}, \end{array}$$ (1)

where *s* = 1, …, *S*_*i*_ indexes the choice situation in which the observation is made, *ε*_*ijs*_ are i.i.d. Gumbel distributed, and *β*_*i*_ ∼ *F*_*β*_(*b*, Σ) is a vector of random coefficients. Estimating the MXL means obtaining the parameters *b* (mean) and Σ (covariance matrix) of the distribution of the *β*’s. The independence of the *β*_*i*_’s implies that the individuals are heterogeneous: their tastes vary following the distribution *F*_*β*_(*μ*, Σ). The correlation between the responses of the same individual to different choice situations, *s* and *s*′, is given by $Cov\left(U_{ijs},U_{iks^{\prime }}\right)=x_{ijs}^{\prime }\Sigma x_{iks^{\prime }}$.

In Eq (1), the coefficients *β*_*i*_ are indexed only by individual, *i*, and not by choice situation, *s*, which means that the tastes of the same individual do not vary between choice situations. This is equivalent to the panel data formulation of MXLs [2]. Specifically, in the route choice problem, travellers’ preferences towards the route attributes are equal for all routes regardless of the OD pair, but the preferences of two different travellers may be different. Also, in the panel data formulation in Eq (1), the alternatives *j* are the same in all the choice situations *s*, i.e., the choice sets are the same across choice situations. This is not true for route choices in several OD pairs, where alternatives are OD pair specific, i.e., $j_{s}\in C\left(s\right)$, where C(s) is the choice set in situation *s*. However, since the variables describing the alternatives are the same for all the choice sets, then the joint mixed logit model can be treated as a MXL for panel data. Conditioning on *β*_*i*_, the joint probability of individual *i* choosing the sequence of alternatives $j_{1},j_{2},\ldots ,j_{S_{i}}$ is given by

$$\begin{array}{c} \begin{array}{cc} L(Y_{i}|\beta_{i}) & =\prod_{s=1}^{S_{i}}\prod_{j=1}^{J}Pr\left(y_{ijs}=1|\beta_{i}\right)\\ & =\prod_{s=1}^{S_{i}}\prod_{j=1}^{J}\frac{\text{exp}(x_{ijs}^{T}\beta_{i})}{\sum_{k\in C(s)}\text{exp}\left(x_{iks}^{T}\beta_{i}\right)}, \end{array} \end{array}$$ (2)

where *y*_*ijs*_ = 1 when alternative $j_{s}\in C\left(s\right)$ is chosen and *y*_*ijs*_ = 0 otherwise; and $Y_{i}={\left(y_{ij1},y_{ij2},\ldots ,y_{ijS_{i}}\right)}^{T}$. The first equality in Eq (2) is guaranteed by the independence of the *ε*_*ijs*_’s, and the second because they are identically Gumbel distributed.

#### Bayesian estimation of panel MXL models

In this work, Bayesian inference is used to estimate *L*(*Y*_*i*_|*β*_*i*_). The classical inference method to estimate the MXL requires integrating the expression in Eq (2) to obtain the unconditional probability *L*(*Y*_*i*_). Nevertheless, since the integral has no closed form, it needs to be numerically approximated, which could present convergence problems and be computationally expensive. An alternative approach to estimate MXLs, is to regard them as Bayesian hierarchical models, which have the advantage of avoiding the numerical multiple integration [2, 38, 39].

In Bayesian methods, the parameters of the model are assumed to be random variables rather than fixed values. Inference, in this context, refers to obtaining the joint distribution of the parameters that best fits the data. To estimate the joint distribution of the parameters, first, a *prior* distribution, *h*, representing the researchers’ beliefs over the values of the parameter, is defined. Then, when data becomes available, the prior is updated through the likelihood function to obtain the *posterior* distribution, *H*. As a result of the Bayes’ theorem, the posterior distribution is proportional to the prior multiplied by the likelihood. In the general case, the posterior distribution *H* of the parameters is

$$\begin{array}{cc} H(b,\Sigma ,\beta_{i},\;\forall i|Y,X)\propto & \\ & [\prod_{i=1}^{N}\prod_{s=1}^{S_{i}}\prod_{j\in C(s)}Pr\left(y_{ijs}=1|x_{ijs};\beta_{i}\right)\phi_{n}\left(\beta_{i}|b,\Sigma \right)]h\left(b,\Sigma \right), \end{array}$$ (3)

where *X* represents the alternative and individuals’ attributes; *Y* the observed choices and *ϕ*_*n*_ is the multivariate normal density function of the random coefficients parametrised by *b* (mean) and Σ (covariance matrix). The expression in brackets is the likelihood of the observed choices and *h* is the joint prior distribution of the model’s parameters. The joint priors, *h*, for the three MXL models estimated in this work, M1, M2 and M3 (see the Results section for the specification of the models), are, respectively,

$$\begin{array}{c} h(b_{p},\sigma_{p}^{2},p=1,\ldots ,5)=\prod_{i=1}^{5}\phi \left(b_{i}|\mu_{0},\sigma_{0}^{2}\right)f_{IG}\left(\sigma_{i}^{2}|r_{0},\lambda_{0}\right)\\ h(b_{p},\sigma_{p}^{2},p=1,\ldots ,6)=\prod_{i=1}^{6}\phi \left(b_{i}|\mu_{0},\sigma_{0}^{2}\right)f_{IG}\left(\sigma_{i}^{2}|r_{0},\lambda_{0}\right)\\ h(b_{p},\Sigma ,\sigma_{p}^{2},p=5,\ldots ,6)=\phi_{4}\left(b_{i=1,\ldots ,4}|\mu_{0},\Sigma_{0}\right)f_{IW}\left(\Sigma |I_{0},k_{0}\right)\\ \;*\phi \left(b_{5}|\mu_{0},\sigma_{0}^{2}\right)f_{IG}\left(\sigma_{5}^{2}|r_{0},\lambda_{0}\right)\phi \left(\beta_{6}|\mu_{0},\sigma_{0}^{2}\right) \end{array}$$

where *ϕ* is the density function of the normal distribution, *ϕ*_*n*_ of the *n*-variate normal distribution, *f*_*IG*_ the density of the inverse-Gamma distribution and *f*_*IW*_ of the inverse-Wishart distribution. The inverse-Gamma is the conjugate prior for the variance of the normal distribution, and the inverse-Wishart its generalisation for the multivariate case.

The right hand side in Eq (3) has no closed form, however samples from the joint posterior distribution *H* can be obtained using the Gibbs sampling method [40]. In this study, the Gibbs sampler software JAGS [41] and the R [42] package *rjags* were used to obtain 10000 samples of the posterior distribution *H* after a burn-in period of 20000 samples. The values of the hyperparameters *μ*_0_, $\sigma_{0}^{2}$, *r*_0_, λ_0_, Σ_0_, *I*_0_ and *k*_0_, which define the priors, were chosen to be weakly-informative (very high variances). In other words, it is assumed high uncertainty on the real values of the parameters that are being estimated. They are shown in Table 1.

**Table 1:** Hyperparameters of the prior distribution *h*.

Hyperparameter	Description
*μ*_0_ = 0; *σ*_0_ = 10, 000	Prior guesses of the mean and variance of the *b* parameter
*r*_0_ = 0.001; λ = 0.001	Prior guesses of the shape and rate of the *σ* parameter
Σ_0*ii*_ = 10, 000, Σ_0*ij*_ = 0 for *i* ≠ *j*	Prior guess of the covariance of the *b* parameter
*I*_0*ii*_ = 4, *I*_0*ij*_ = 0, *i* ≠ *j*; *k*_0_ = 4	(1/*k*_0_)*I*_0_ is the prior guess of the covariance Σ

## Results

### Clustering

**Fig 3:**
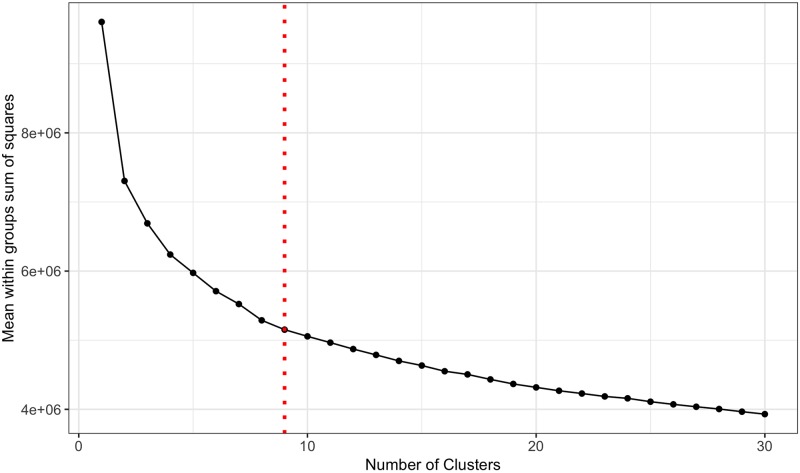
Determining the number of clusters *k*. The sum of squared errors for *k*-means clustering of the 624,490 OD-routes with *k* = 1, …, 30. After *k* = 9 the decrease in the mean WCSS is marginal.

**Fig 4:**
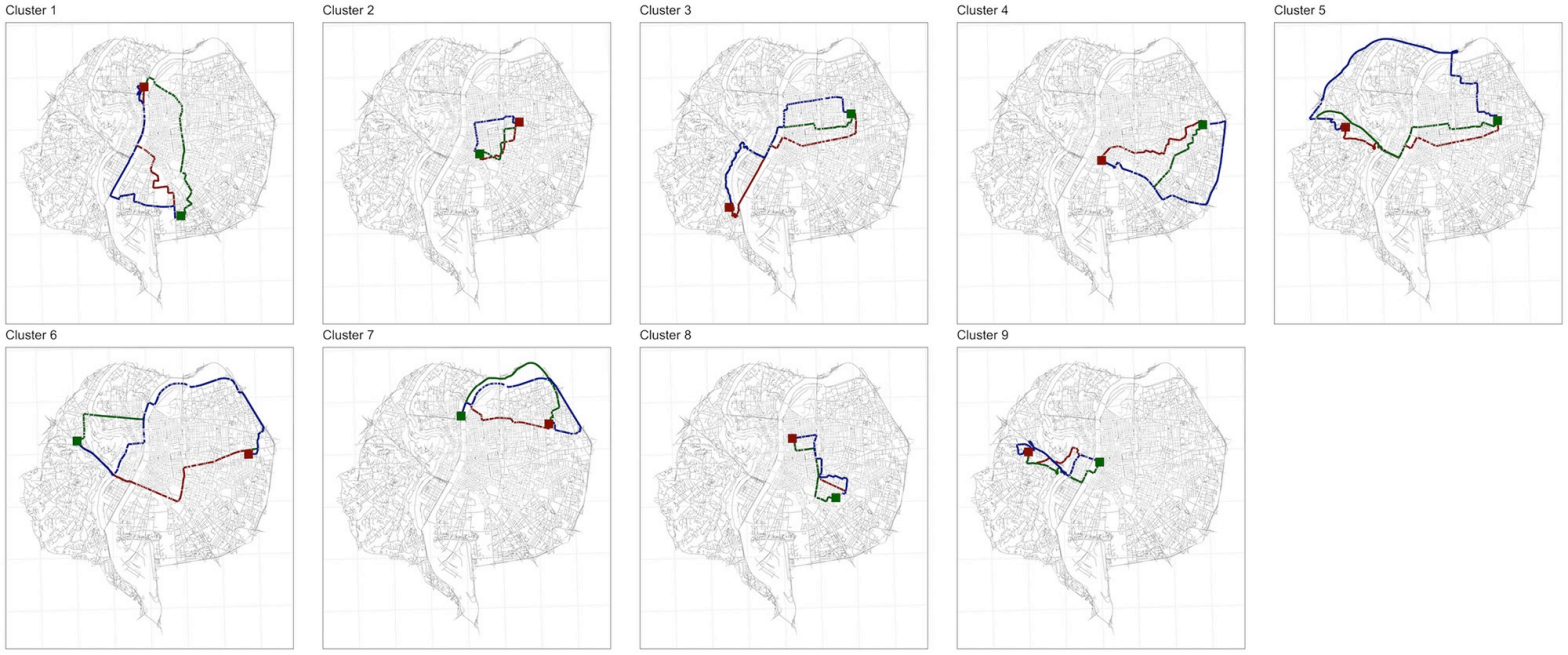
Selected OD-routes for the route choice experiments. The geodata used to render the plot is from ©OpenStreetMap contributors, licensed under the Open Data Commons Open Database License (ODbL). The maps were rendered using The R Project for Statistical Computing which is distributed as Free Software under the terms of the Free Software Foundation’s GNU General Public License.

To determine the optimal number of clusters, *k*-means algorithm, with *k* = 1, …, 30, was performed over the 624,490 OD-routes in the network. The mean within-cluster sum of squares (WCSS) is plotted against the number of clusters *k* in Fig 3. In the results, the optimal number of clusters is not clear, according to the elbow method: big improvements happen for the first values of *k* (*k* ≤ 4); for values 5 ≤ *k* ≤ 9 the improvement is mediocre; and for *k* ≥ 10 the improvements are rather small. In terms of the purpose of this article, choosing a small number of *k* has the risk of sub-representing the OD-routes in the network and, more important, a small number of OD-routes in the route choice experiments implies that the variability in the route attributes is also small, posing a problem in estimating a route choice model (overfitting). In this sense, choosing high values of *k* is preferable, even if some of the clusters are similar. However, the needed number of observations in the route choice experiment increases with the number of OD-routes, implying higher costs in the organisation of the experiments, not to mention the difficulties to recruit participants. In view of these limitations, the number of clusters is set to *k* = 9. The clustering results are presented in Table 2 and the centroids are depicted in the map shown in Fig 4.

**Table 2:** Cluster analyis results.

Cluster	1	2	3	4	5	6	7	8	9	Total
**No. obs**	62,479	86,004	60,063	63,101	44,130	49,956	53,036	119,557	86,164	624,490
**WCSS**	564,188	578,003	569,158	527,322	464,730	491,656	535,509	759,205	663,009	5,152,782
**Variance**	9.03	6.72	9.48	8.36	10.5	9.84	10.1	6.35	7.69	15.38

**Fig 5:**
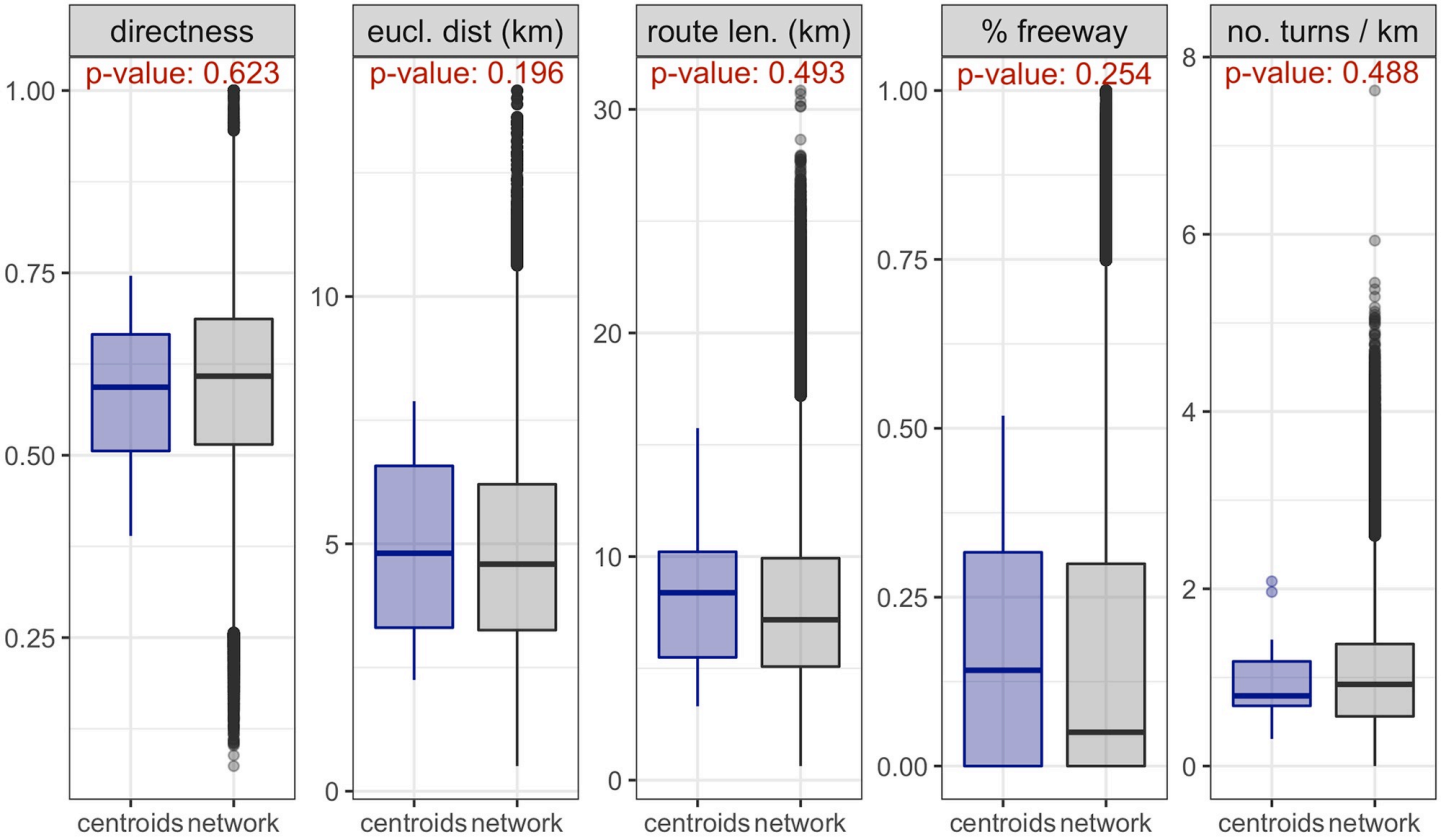
Cluster centroids as representative OD-routes. The distribution of the attributes of the selected OD pairs are similar to that of the whole network. The p-values of the Kolmogorov-Smirnov, presented in red in the top of each panel, indicate the lack of statistical evidence (with a confidence level of 0.90) to reject the hypothesis that the two distributions are the same.

With *k* = 9, the variability of the full set of OD-routes is reduced in 46.4%. If well, this reduction may not be big in terms of clustering analysis, it can be seen (Fig 5) that the road attributes of the cluster centroids cover likely values to be observed in the network. To be more specific, 83% of the values of the attributes of the OD-routes in the network lie in the range of the centroids: 83% for the euclidean distance and the directness, 89% for the freeway composition, 85% for the number of turns per kilometre and 90% for the route length. Furthermore, the resulting p-values of the two-sample Kolmogorov-Smirnov test (Fig 5) are high: *p* − *value* > 0.1 for the five variables, suggesting that there is not enough statistical evidence (with a significance level of *α* = 0.1) to reject the null hypothesis that the values of the attributes of the centroids and the full network come from the same distribution. This implies that a random selected OD pair or route in the network is likely to have attributes similar to one of the nine OD-routes used in the route choice experiments. In this sense, the nine cluster centroids can be regarded as representative of the network.

A further characterisation of the nine clusters, based on their elements’ attributes (see S1 and S2 Figs), is proposed as follows:

**Clust**. *C*_1_: Medium-range direct trips going from south to north, with routes having small number of turns per kilometre and some freeway segments.
**Clust**. *C*_2_: Short non direct trips mainly in the central part of the network, with routes having a lot of turns per kilometre and no freeway segments.
**Clust**. *C*_3_: Medium-range direct trips going from north to south, with routes having small number of turns per kilometre and some freeway segments.
**Clust**. *C*_4_: Medium-range direct trips mostly in the central part of the network, with routes having average number of turns per kilometre and with longest route highly composed of freeway segments.
**Clust**. *C*_5_: Long trips going from east to west, with routes having a small number of turns and with large portions of freeway.
**Clust**. *C*_6_: Long trips going from west to east, with routes having a small number of turns and with large portions of freeway.
**Clust**. *C*_7_: Medium-range non direct trips in the central part of the network, with routes with average number of turns per kilometre and high portions of freeway.
**Clust**. *C*_8_: Short direct trips mainly in the central part of the network with routes with low number of turns per kilometre (among short trips) and no freeway segments.
**Clust**. *C*_9_: Short non direct trips mainly in the central part of the network, with routes with low number of turns per kilometre (among short trips) and some freeway segments.

### Experiment results

**Fig 6:**
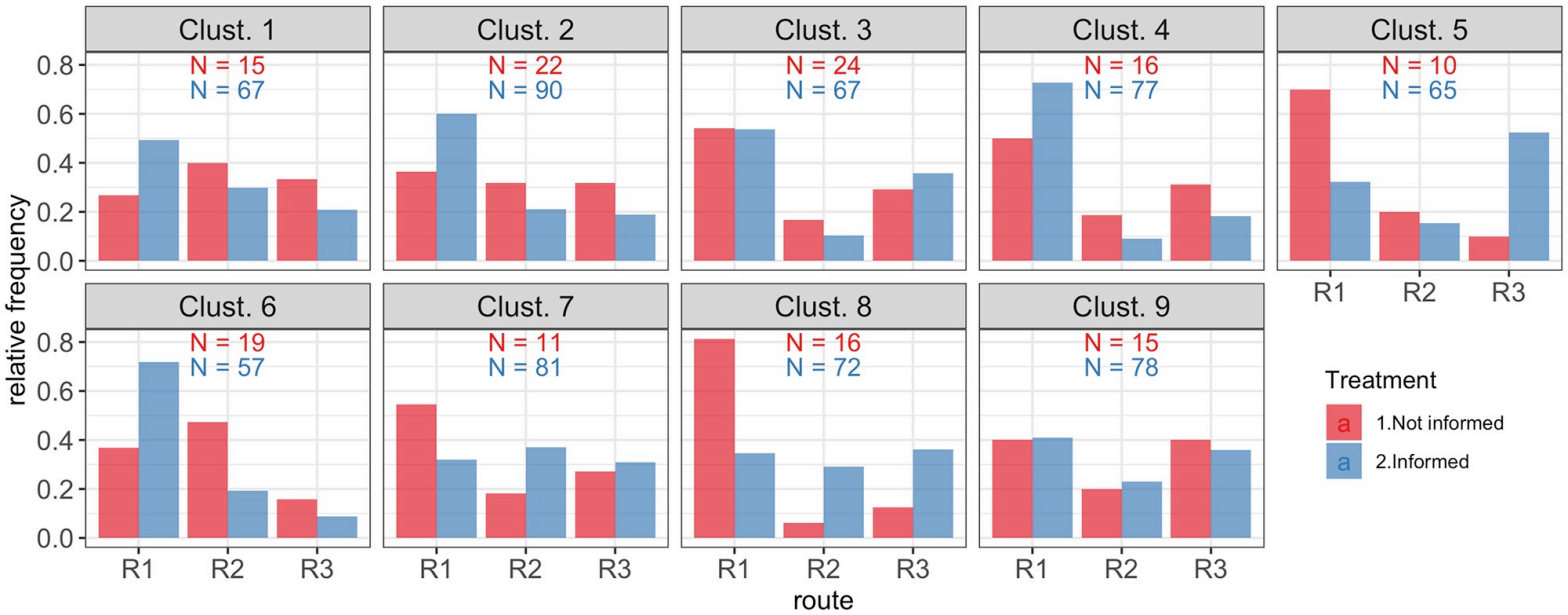
Route choice distribution in the nine cluster centroids. The choices of the informed participants are different from those of the not informed participants.

Three route choice experiment sessions were carried out using the nine OD pairs and routes obtained from the clustering analysis of the network. In total, 73 individuals participated in the three sessions, from these participants, 56 (77%) received estimates of the travel times in each route. Participants recorded a total number of 802 choices in the nine defined OD-routes, with an average number of 11 choices per participant, and an average number of 89 choices in each OD-routes. The choices of the participants are presented in Fig 6, where it can be immediately noticed that travel time information changes the behaviour of the participants.

### Route choice model estimation

Three panel data mixed logit models are estimated using the observations collected in the route choice experiments. Five variables are used in the specification of the models. Four of these variables correspond to the variables used in the selection of the representative OD-routes for the route choice experiments, which will help to test if the choices in the representative OD-routes (cluster centroids) can approximate the choices in other OD-routes. The fifth variable is the estimated travel time that the participants received during the experiments. These variables are known to influence the route choice behaviour of travellers and that can be observed by the participants in the computer route choice experiments.

Let the individuals and alternatives be indexed by *i* and *j*, respectively. Since participants were allowed to repeat decisions in the same OD pair, the choice situation, indexed by *s*, represents the pair (*od*, *t*), where *od* is the OD pair in which the decision was made and *t* indexes the moment of the choice. The explanatory variables considered in the model are

- *FRW*_*j*_, the percentage of freeway that composes the route *j*;
- *DIR*_*j*_, the directness of the route *j*, defined as the length of *j* divided by the euclidean distance between origin and destination;
- *TNR*_*j*_, the number of turns per kilometre in the route *j*;
- *ITT*_*js*_, the informed travel time in the route *j* in OD pair and moment *s*, the variable is normalised by OD pair by dividing the informed travel time by the free flow travel time in the fastest of the three routes;
- *INF*_*i*_, binary variable indicating if participant *i* received information; and
- *LEN*_*j*_, the length (in km) of the route *j*.

The specifications of the three models M1, M2 and M3 are

$$\begin{array}{c} U_{ijs}=\beta_{i1}FRW_{j}+\beta_{i2}DIR_{j}+\beta_{i3}TNR_{j}+\beta_{i4}LEN_{j}+\beta_{i5}ITT_{js}INF_{i}+\varepsilon_{ijs}\;, \end{array}$$ (M1)

$$\begin{array}{c} U_{ijs}=\beta_{i1}FRW_{j}+\beta_{i2}DIR_{j}+\beta_{i3}TNR_{j}+\beta_{i4}LEN_{j}+\\ \;\beta_{i5}ITT_{js}INF_{i}+\beta_{i6}ITT_{js}LEN_{j}INF_{i}+\varepsilon_{ijs}\; \end{array}$$ (M2)

$$\begin{array}{c} U_{ijs}=\beta_{i1}FRW_{j}+\beta_{i2}DIR_{j}+\beta_{i3}TNR_{j}+\beta_{i4}LEN_{j}+\\ \;\beta_{i5}ITT_{js}INF_{i}+\beta_{6}ITT_{js}LEN_{j}INF_{i}+\varepsilon_{ijs}\;. \end{array}$$ (M3)

In models M1 and M2, the coefficients *β*_*ip*_ for *p* = 1, ‥, 6 are independent and normally distributed, i.e., $\beta_{ip}∼N\left(b_{p},\sigma_{p}^{2}\right)$. In model M3, the coefficient *β*_6_ is fixed for all individuals (not random), and the coefficients *β*_*ip*_ are correlated for *p* = 1, ‥, 4, i.e., *β*_*i*._ ∼ *N*_4_(*b*, Σ), but independent from $\beta_{i5}∼N\left(b_{5},\sigma_{5}^{2}\right)$. Model M1 is the simplest MXL model considering the five variables. In models M2 and M3 the interactions between the route length and the travel time information are taken into account, allowing for the preference towards the length of the route to change depending on the informed travel time. In model M3 the correlations between the coefficients *β*_*ip*_ for *p* = 1, …, 4 are also estimated. In MXL models, the parameters that are estimated are the means and variances (covariances) of the coefficients’ distributions, $\hat{b_{p}}$, $\hat{\sigma }$ and $\hat{\Sigma }$. The estimated parameters for the three models are shown in Table 3; more detailed result of the posterior distribution of the parameters can be found in S1 Table, and the details of the computational effort for the estimation process in S2 Table.

**Table 3:** MXL models estimation results.

Coefficient	M1	M2	M3
$\hat{b_{1}}$ (*FRW*_*j*_)	1.96 (0.85)	2.01 (0.74)	2.11 (0.80)
$\hat{b_{2}}$ (*DIR*_*j*_)	4.61 (1.65)	4.00 (2.10)	4.56 (1.87)
$\hat{b_{3}}$ (*TNR*_*j*_)	-0.15 (0.26)	-0.14 (0.26)	-0.20 (0.30)
$\hat{b_{4}}$ (*LEN*_*j*_)	0.01 (0.12)	-0.11 (0.13)	-0.14 (0.16)
$\hat{b_{5}}$ (*ITT*_*js*_)	-3.86 (0.85)	-4.58 (1.01)	-5.28 (1.24)
$\hat{b_{6}}$ (*ITT*_*js*_ * *LEN*_*j*_)	-	0.08 (0.06)	0.13 (0.10)
$\hat{\sigma_{1}}$ (*FRW*_*j*_)	0.61 (0.61)	0.58 (0.62)	2.33 (1.15)
$\hat{\sigma_{2}}$ (*DIR*_*j*_)	1.10 (1.00)	0.88 (0.98)	3.00 (2.66)
$\hat{\sigma_{3}}$ (*TNR*_*j*_)	0.72 (0.39)	0.76 (0.40)	1.24 (0.29)
$\hat{\sigma_{4}}$ (*ITT*_*js*_)	0.11 (0.05)	0.10 (0.06)	0.51 (0.13)
$\hat{\sigma_{5}}$ (*ITT*_*js*_)	4.62 (0.87)	4.64 (0.90)	4.72 (0.91)
$\hat{\sigma_{6}}$ (*ITT*_*js*_ * *LEN*_*j*_)	-	0.07 (0.04)	-
$\hat{\sigma_{12}}$ (*FRW*_*j*_-*DIR*_*j*_)	-	-	1.96 (8.73)
$\hat{\sigma_{13}}$ (*FRW*_*j*_-*TNR*_*j*_)	-	-	1.21 (1.60)
$\hat{\sigma_{14}}$ (*FRW*_*j*_-*ITT*_*js*_)	-	-	-0.36 (0.65)
$\hat{\sigma_{23}}$ (*DIR*_*j*_-*TNR*_*j*_)	-	-	0.63 (2.28)
$\hat{\sigma_{24}}$ (*DIR*_*j*_-*ITT*_*js*_)	-	-	0.83 (2.06)
$\hat{\sigma_{34}}$ (*TNR*_*j*_-*ITT*_*js*_)	-	-	0.00 (0.19)

Mean (standard deviation) of the sampled posterior distributions of the parameters of the MXL models.

The estimated parameters $\hat{b_{p}}$ represent the mean preferences in the population. The positive sign of the estimates $\hat{b_{1}}$ and $\hat{b_{2}}$ in the three models is interpreted as *the average traveller prefers routes with high composition of freeways, and direct routes*. On the contrary, the negative signs of $\hat{b_{3}}$ and $\hat{b_{5}}$ mean that *the average traveller avoids routes with many turns and higher travel times*. These results are in line with the findings in [24], and provide more evidence in favour of travel time as the most important variable in route choice. Note that in the three models $\hat{b_{3}}\approx 0$, but with large standard deviations $\hat{\sigma_{3}}$, meaning that (i) the sign is positive for a large number of participants (near half), and that (ii) even when the mean of the coefficient is close to zero, this variable is still important for a large percentage of the participants, specially in model M3, where *Pr*(|*β*_*i*3_| > 1) = 0.42. The case for the length of the route is different, as the standard deviations are smaller: for models M1 and M2 this implies that the length of the route is not important for the majority of the participants, *Pr*(|*β*_*i*4_| < 0.2) = 0.93 and *Pr*(|*β*_*i*4_| < 0.2) = 0.81, respectively; but for model M3 it is, *Pr*(|*β*_*i*4_| < 0.2) = 0.29. Finally, note that in models M2 and M3 the mean preference for the length of the routes can be written as (*b*_4_ + *b*_6_*ITT*_*js*_ *INF*_*i*_), with *b*_4_ < 0 and *b*_6_ > 0, meaning that the informed travel time diminishes the preference for shorter routes.

### Choices on representative OD-routes

Until now, the discussion on the representativeness of the nine selected OD-routes (the cluster centroids) has been in terms of the route attributes. In this section, the representativeness of the OD-routes is assessed in terms of how well a choice model, estimated using the cluster centroids, can be generalised to the entire road network or, in other words, how well it scales-up the travellers’ choices to other OD pairs in the network. The hypothesis is that if the choices in the nine cluster centroids are representative of the choices in the entire network, then the predictive accuracy of a model, estimated with observations in the nine cluster centroids, should be higher than the predictive accuracy of models (with the same specification) estimated with observations in random sets of OD-routes. To this end, data collected in other route choice experiments carried out with the MDG platform is used. The data consists of route choice observations in 21 OD-routes, defined with a different methodology for previous experiments, and not comprising the representative OD pairs.

The methodology to validate the representative OD-routes is based on bootstrapping for model validation: at each step, a random part of the data is left-out of the estimation process, and then used to measure the predictive accuracy of the model. However, in this case, the predictive accuracy of the models obtained at each iteration are compared to the predictive accuracy of the model estimated with the nine cluster centroids. Let *C* be the set of choice observations in the 9 cluster centroids and *T* the set of observations in the 21 test OD-routes. Denote by M* the model (it can be either M1, M2 or M3) estimated with observations on the nine cluster centroids, *C*. At iteration *r*, *r* = 1, …, 40,

1. obtain *T*_*r*_ ⊂ (*C* ∪ *T*), composed of all the observations from nine randomly sampled OD-routes;
2. estimate the model *M*_*r*_ (M1, M2 or M3) with the observations in *T*_*r*_;
3. for each OD-routes *od* ∈ (*C* ∪ *T*) − *T*_*r*_, compute the prediction error of models M* and *M*_*r*_, i.e., *e*_*r*_(M*, *od*) and *e*_*r*_(*M*_*r*_, *od*), where
  $$\begin{array}{c} e_{r}\left(M^{*},od\right)=\sum_{i=1}^{3}max\left(0,obs_{i}-pred_{i}\right)\;; \end{array}$$ (4)
4. obtain the mean prediction error for iteration *r*, defined as
  $$\begin{array}{c} MPE_{r}\left(M_{r}\right)=\sum_{od\in T_{r}}w_{od}e_{r}\left(M_{r},od\right)\;, \end{array}$$ (5)
  where the weight *w*_*od*_ is the percentage of OD-routes in the cluster to which *od* belongs, multiplied by the inverse of the number of OD-routes in (*C* ∪ *T*) that belong to that cluster. The weighting is done to adjust for the probability of observing an OD-routes in the network like *od*. This follows since some clusters are over-represented in *T*, as the OD-routes in *T* were not randomly selected from the network, but they were selected following a different methodology in previous studies.

Note that the error measure, *e*_*r*_(M*, *od*) has a direct interpretation in terms of traffic assignment: the percentage of trips that are wrongly distributed amongst the three alternative routes.

**Fig 7:**
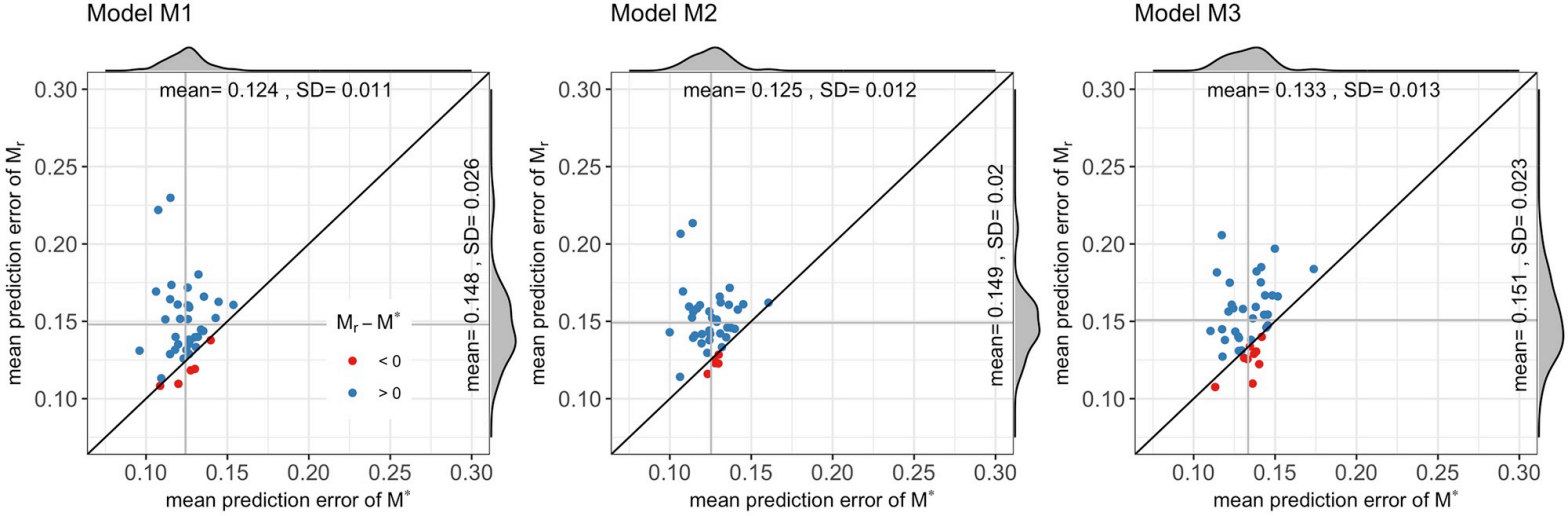
Mean predictive errors. The MPEs of model M* are smaller than the MPEs of models *M*_*r*_ in the majority of the cases (blue dots). Furthermore, in the cases where the MPEs of models M* are bigger (red dots), the differences are small (close to identity line).

The *MPE*_*r*_(M*) is compared against the *MPE*_*r*_(*M*_*r*_), *r* = 1, …, 40 for the three model specifications. In Fig 7, *MPE*_*r*_(M*) is plotted against *MPE*_*r*_(*M*_*r*_), with blue dots when *MPE*_*r*_(M*) ≤ *MPE*_*r*_(*M*_*r*_), and red otherwise. The models estimated with the clusters’ centroids performed better in predicting the choices of travellers than most of the models estimated with randomly selected OD-routes. To be more specific, *MPE*_*r*_(M*) ≤ *MPE*_*r*_(*M*_*r*_) in 35 out of 40 cases (87.5%) for models M1 and M2, and in 31 cases (77.5%) for model M3. Furthermore, in the cases when the model estimated with the centroids performed worst, i.e., *MPE*_*r*_(M*) ≤ *MPE*_*r*_(*M*_*r*_), the errors were close to those of the models estimated with randomly selected OD-routes. Define the improvement of M* with respect to *M*_*r*_ as *α*_*r*_ = (*MPE*_*r*_(*M*_*r*_) − *MPE*_*r*_(M*))/*MPE*_*r*_(*M*_*r*_). Then, the mean improvements, $\bar{\alpha }$, are 14% for model M1, 14.5% for model M2 and 9.9% for model M3. In 20% of the test cases, *α*_*r*_ is at least 26%, 25% and 22% for models M1, M2 and M3, respectively; and *α*_*r*_ reaches 52%, 48% and 43% in the worst case scenarios. This results highlights the importance of a careful selection of OD pairs in route choice model estimation. As the MPE represents the percentage of trips that are not assigned to the right route, and since the total number of trips at a city level can be very high, about 1 million in the Lyon Metropolis during one day, even low *α*_*r*_ values may have an impact on how the traffic is distributed on the network.

**Fig 8:**
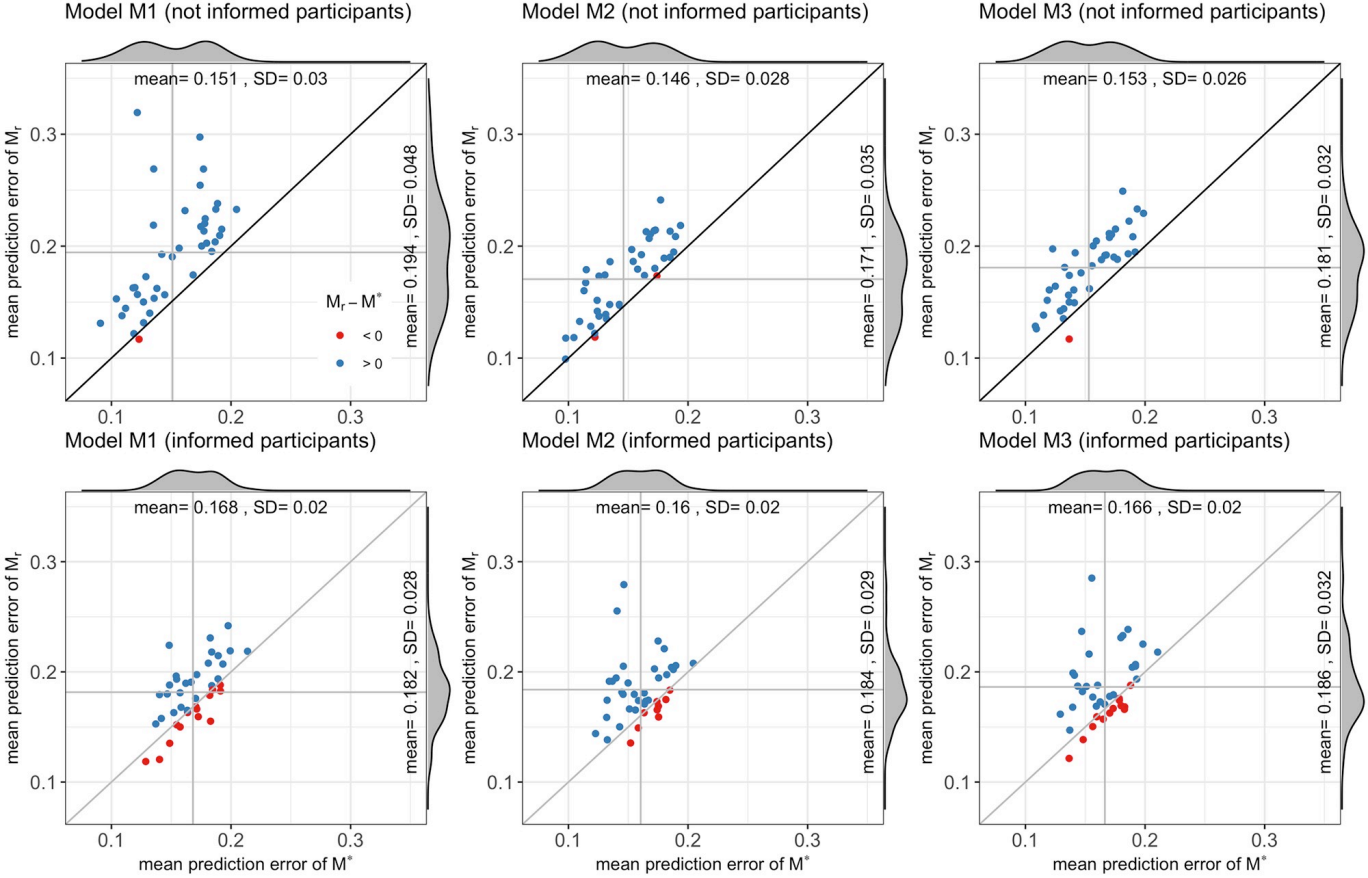
Mean predictive errors by information group. The models estimated with the cluster centroids are clearly better in predicting the choices for the not informed participants.

If the MPE is analysed by whether or not the participants received travel time information (Fig 8), it can be seen that the models M* are better than the models *M*_*r*_ for the not informed participants than for the informed ones. For models M1 and M3, *MPE*_*r*_(M*) ≤ *MPE*_*r*_(*M*_*r*_) in 97.5% of the cases, and for model M2 in 95%; and when the participants were informed, *MPE*_*r*_(M*) ≤ *MPE*_*r*_(*M*_*r*_) in 65% for model M1, 77.5% for model M2 and 67.5% for M3. In the case of the not informed participants the values of $\bar{\alpha }$ are 20%, 14% and 15%, respectively for models M1, M2 and M3. The high performance of the models M* for the not informed participants implies that the models are capable of approximating the choices of this group in a variety of scenarios, i.e., the models estimated with the nine centroids generalise well to other OD-routes for this group. Moreover, considering that the informed travel time was not part of the variables used in the clustering of the OD-routes (% of freeway, directness, no. of turn per kilometre, distance), this result suggests that the choices in the cluster centroids are representative of the choices in the entire network, thus validating the methodology proposed in this article.

**Fig 9:**
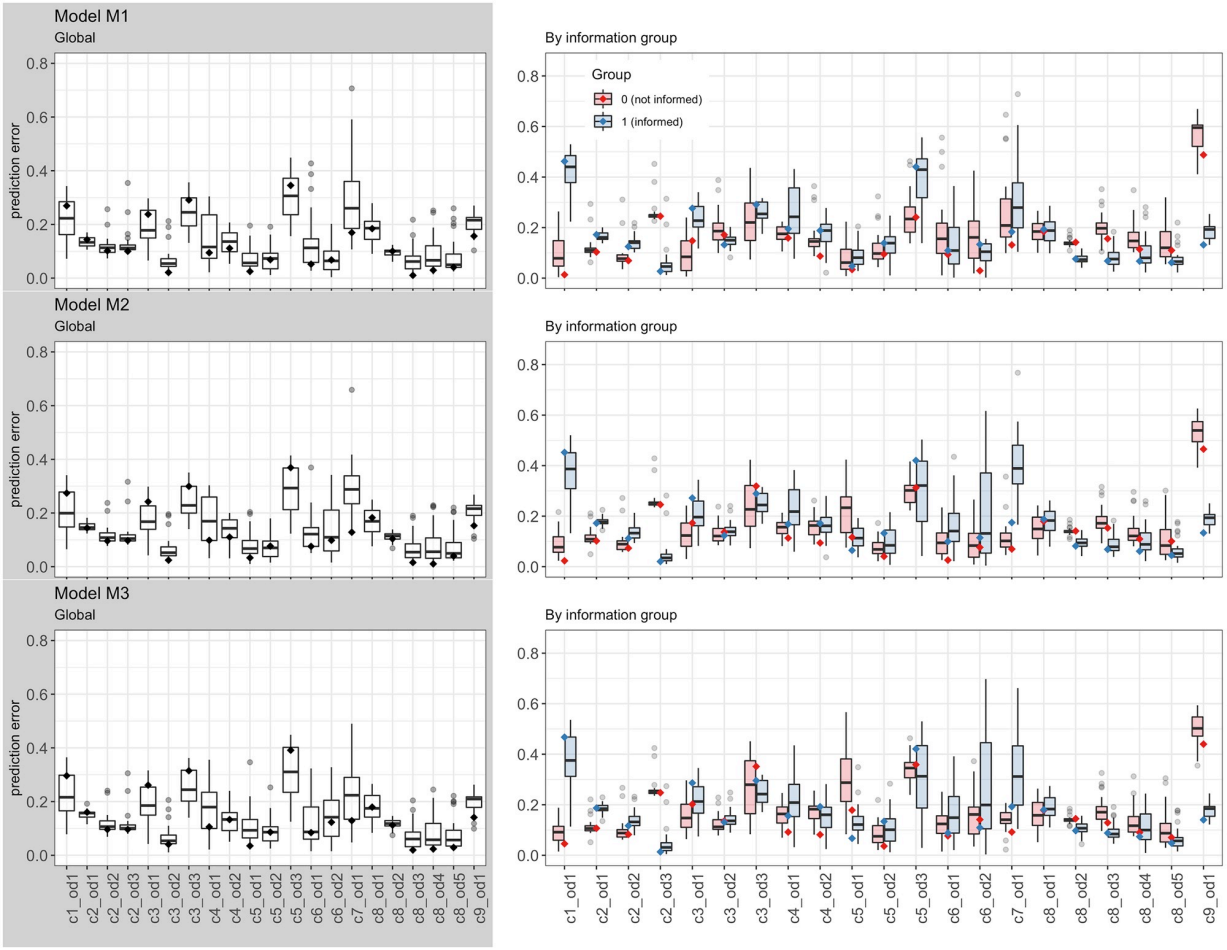
Distributions of the predictive errors the 21 validation OD-routes. The level and the variability of the errors amongst the different OD-routes imply that the choices in some OD-routes are difficult to predict, regardless of the training set used to estimate the models.

The predictive errors of the representative models, M*, and the test models, *M*_*r*_, can be disaggregated by OD-routes. In the results, shown in Fig 9, it is clear that the magnitude and the variance of the predictive errors depend on the OD-routes where the choices are being predicted. The choices in some OD-routes are difficult to predict, regardless of the training set used to estimate the models. Furthermore, there is no clear pattern indicating that these errors are associated with the road characteristics of the OD-routes: two OD-routes belonging to the same cluster, i.e., having similar route attributes, may have a low and a high prediction error. Such is the case of OD-routes c3_od2 and c3_od3, both belonging to cluster *C*_3_, but with errors below 0.1 for the former and above 0.2 for the later. Similar cases can be found in cluster *C*_5_ and *C*_8_. An important observation is that the models estimated with the cluster centroids M* are not as accurate in predicting the choices in individual OD-routes as the models *M*_*r*_, for some values of *r*. In fact, their prediction errors are amongst the lowest 25% in only 8 out of 21 test OD-routes for models M1 and M3, and in 6 for model M2. However, at the same time, the individual errors are almost never amongst the highest 75%: in 0 OD-routes for model M1, in 2 for model M2, and in 1 for M3. Moreover, when the individual errors are averaged to obtain the MPE (as in the previous analysis), the models M* outperform the models *M*_*r*_ for the majority of values of *r*. This result implies that a model $M_{r_{0}}$ having low prediction errors for some OD-routes has also high prediction errors in other OD-routes, and therefore its mean predictive accuracy is reduced. In this sense, the models estimated with the cluster centroids, M*, are preferred, as they will show a relative better global prediction accuracy without incurring in large errors in individual OD-routes.

**Fig 10:**
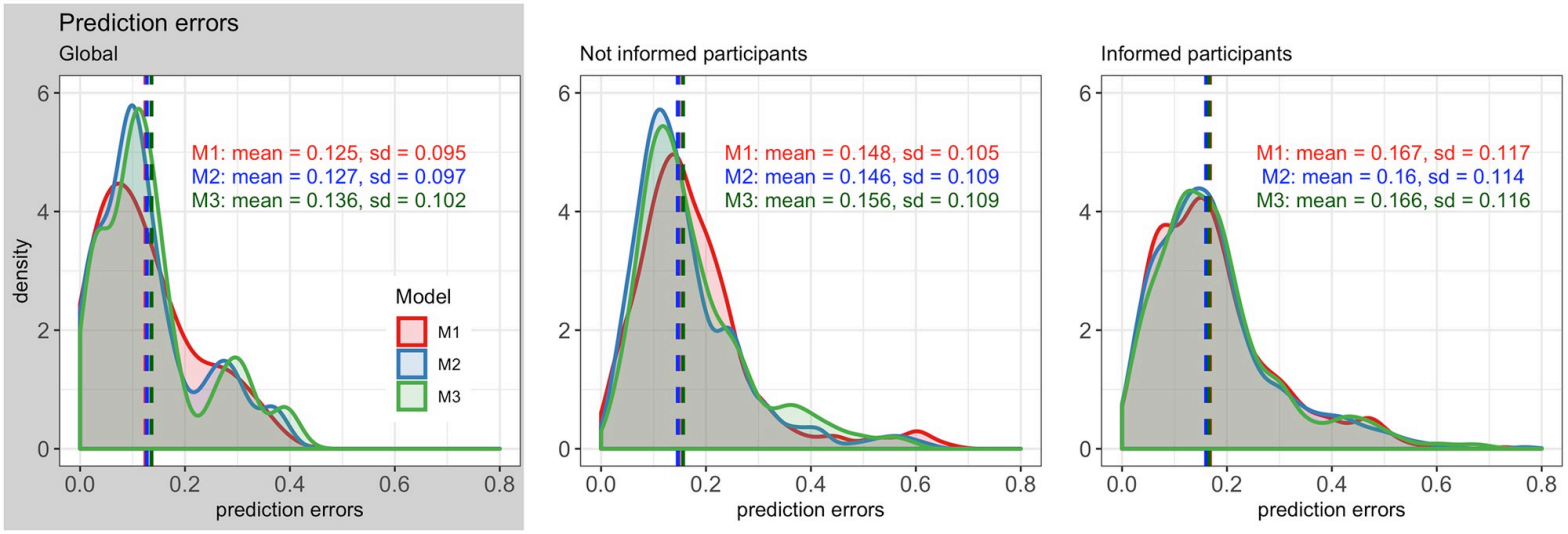
Distributions of the prediction errors of the models M* on the 21 validation OD-routes. There are no significant differences between the error distributions.

The models estimated with the representative OD-routes, M*, are compared in terms of their prediction errors over the 21 validation OD-routes. The error distribution of the three models, depicted in Fig 10, show that, practically, there is no difference in the predictive accuracy. This means that the interaction between the informed travel time and length of the route in models M2 and M3 does not improve the predictive accuracy; nor considering the correlations in model M3 does.

## Discussion

In this study, it was demonstrated that the choices of participants in a route choice experiment over a small but representative set of OD configurations can be scaled-up to the entire network. To obtain the set of representative OD configurations, a new methodology based on *k*-means cluster analysis is proposed. First, the OD configurations in the network, i.e., the OD pairs and three connecting routes, are represented in vector form according to the attributes of the OD pairs and routes. Then, these OD configurations are clustered in order to obtain a partition of the road network and the cluster centroids selected as representative of the entire network. The main hypothesis is that the choices of travellers over the entire network can be approximated with route choice models estimated using data collected for the representative set. The obtained results point in this direction.

In the current study, for the city of Lyon in France, 9 OD pairs and their connecting routes were used as representative of 624,490 OD configurations. These nine representative OD configurations cover around 83% of the values of the attributes of the OD-routes in the network. The predictions of the models estimated with the representative set were superior in most of the test cases (87.5% and 77.5% in the general case). For the not informed participants, whose decisions were based on the same attributes used in the clustering, the predictions are better in at least 95% of the test cases. By estimating the route choice model with the cluster centroids, the mean prediction errors are reduced by up to 14.5% for model M1 (similar results are observed for models M2 and M3). The reduction of the prediction error is more than 22% for the 20% of the test cases, and it goes up to 51% in the worst case. This demonstrates that a careful selection of the OD configurations significantly improves the prediction accuracy, independently of the model specification. Another significant finding, is that the models estimated with the representative OD configurations are more robust than the ones obtained from the models with random OD configurations. The models estimated with the representative set never show extreme errors for individual OD pairs, contrary to the models estimated with random sets of OD configurations. This implies that the models estimated with the representative set will show a relative better global prediction accuracy without incurring in large errors on individual OD-routes. This result is important when predicting the trip distribution over the network, as high errors in individual OD pairs may have significant impact in local traffic conditions, causing spreading.

The last finding is that estimating the models with the representative OD pairs leads to an average prediction error of 12.7%. This value can be considered quite low when considering the scale of the city, the heterogeneity of OD configurations, and the actual performance of user equilibrium approaches.

From the clustering analysis in this study, it is clear that there are OD pairs in the network that are not well represented by the representative set of nine OD configurations. Therefore, it cannot be claimed that the choices in these non-represented OD pairs can be well approximated by the set of nine OD pairs found in this study. However, these non-represented OD pairs are those with attributes not covered by the representative set, which are no more than 17% of the OD configurations in the network. Note that this result does not hinder the usefulness of the proposed methodology, as it can be extended by either using other clustering techniques that allow taking into account for these *atypical* OD configurations or by including more clusters in the representative set.

## Supporting information

S1 Fig Distribution of the route attributes in the nine clusters. (TIF)

S2 Fig Origins and destinations in the network. (TIF)

S1 Table Complete estimation results of the MXL models. (PDF)

S2 Table Computational effort in the estimation of the MXL models. (PDF)
