# Supplementary figures and images for: Unravelling travellers’ route choice behaviour at full-scale urban network by focusing on representative OD pairs in computer experiments
Source: PLoS One. 2019 Nov 12;14(11):e0225069. doi: 10.1371/journal.pone.0225069 (PMC6850682; doi:10.1371/journal.pone.0225069)

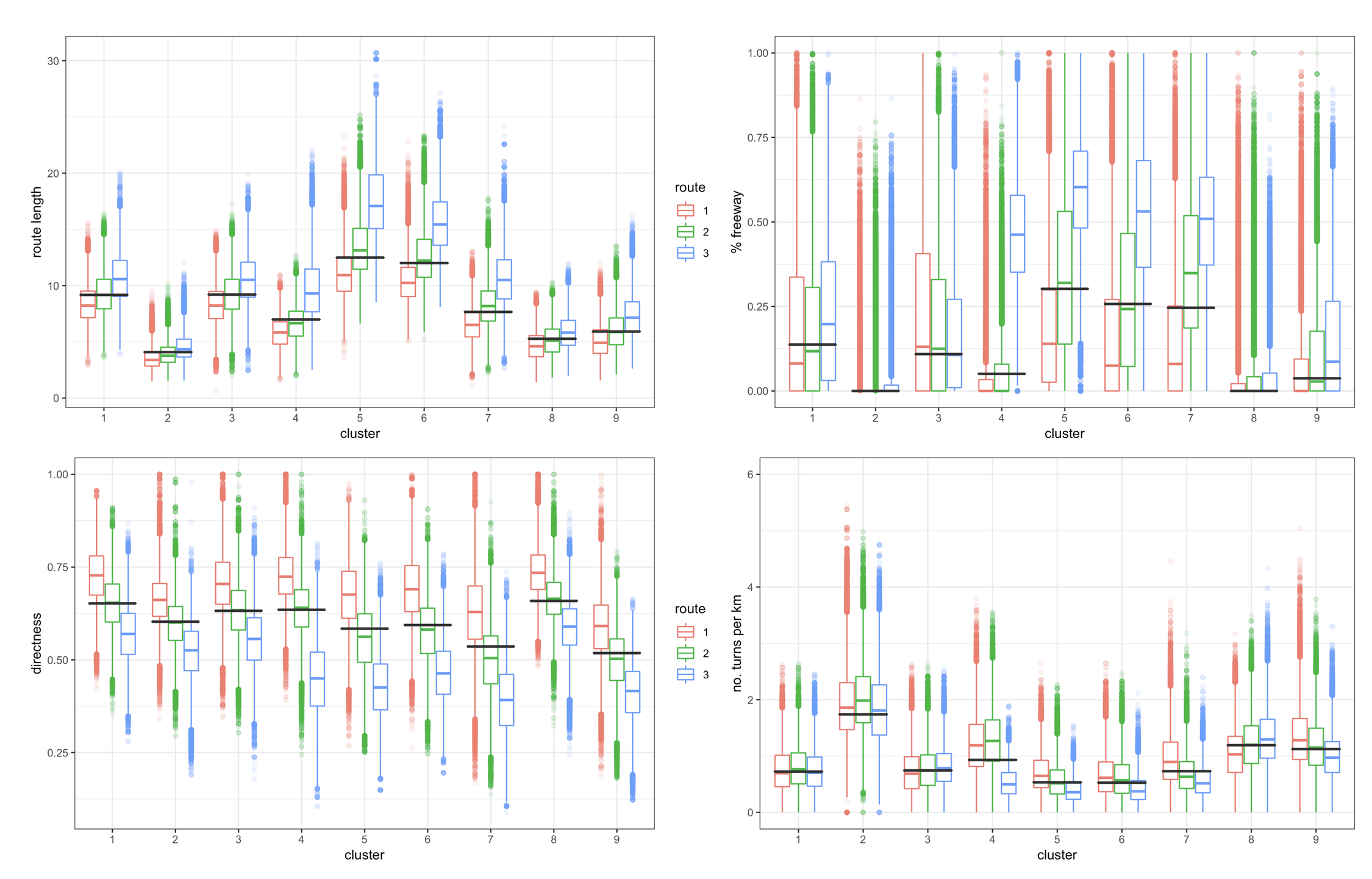

Supplement: S1 Fig — (TIF) [file pone.0225069.s001.tif]

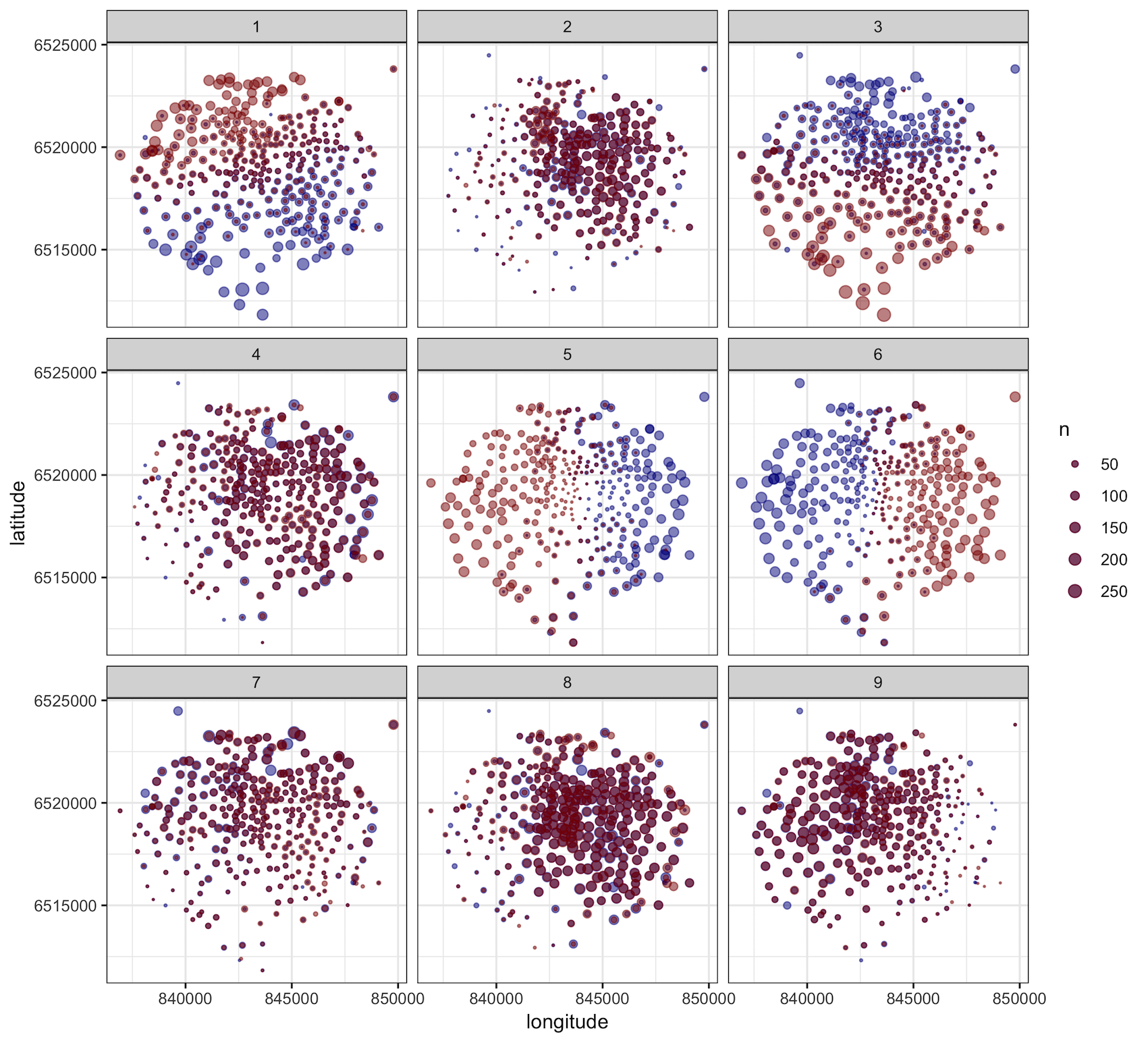

Supplement: S2 Fig — (TIF) [file pone.0225069.s002.tif]
