# Supplementary material for: Unravelling travellers’ route choice behaviour at full-scale urban network by focusing on representative OD pairs in computer experiments
Source: PLoS One. 2019 Nov 12;14(11):e0225069. doi: 10.1371/journal.pone.0225069 (PMC6850682; doi:10.1371/journal.pone.0225069)
Supplement: S1 Table — (PDF) [file pone.0225069.s003.pdf]

Complete estimation results of the MXL models

Coefficient	mean	s.d.	q. 2.5%	q. 25%	q. 50%	q. 75%	q. 97.5%
**M1**							
$\hat{b_1}$ (FRW_j)	1.96	0.85	0.44	1.31	1.92	2.67	3.47
$\hat{b_2}$ (DIR_j)	4.61	1.65	1.22	3.56	4.64	5.73	7.55
$\hat{b_3}$ (TNR_j)	-0.15	0.26	-0.65	-0.33	-0.15	0.02	0.36
$\hat{b_4}$ (LEN_j)	0.01	0.12	-0.26	-0.07	0.02	0.09	0.22
$\hat{b_5}$ (ITT_{js})	-3.86	0.85	-5.61	-4.42	-3.85	-3.29	-2.23
$\hat{\sigma_1}$ (FRW_j)	0.61	0.61	0.04	0.15	0.38	0.88	2.19
$\hat{\sigma_2}$ (DIR_j)	1.10	1.00	0.04	0.20	0.85	1.75	3.43
$\hat{\sigma_3}$ (TNR_j)	0.72	0.39	0.05	0.43	0.74	1.00	1.46
$\hat{\sigma_4}$ (ITT_{js})	0.11	0.05	0.03	0.07	0.10	0.14	0.23
$\hat{\sigma_5}$ (ITT_{js})	4.62	0.87	3.12	4.00	4.55	5.15	6.52
**M2**							
$\hat{b_1}$ (FRW_j)	2.01	0.74	0.69	1.47	1.96	2.57	3.41
$\hat{b_2}$ (DIR_j)	4.00	2.10	0.74	2.34	3.85	5.80	7.81
$\hat{b_3}$ (TNR_j)	-0.14	0.26	-0.64	-0.32	-0.15	0.03	0.35
$\hat{b_4}$ (LEN_j)	-0.11	0.13	-0.32	-0.21	-0.12	-0.01	0.13
$\hat{b_5}$ (ITT_{js})	-4.58	1.01	-6.60	-5.25	-4.57	-3.91	-2.64
$\hat{b_6}$ ($ITT_{js} * LEN_j$)	0.08	0.06	-0.04	0.04	0.08	0.12	0.21
$\hat{\sigma_1}$ (FRW_j)	0.58	0.62	0.03	0.11	0.31	0.91	2.15
$\hat{\sigma_2}$ (DIR_j)	0.88	0.98	0.03	0.11	0.44	1.41	3.41
$\hat{\sigma_3}$ (TNR_j)	0.76	0.40	0.05	0.48	0.79	1.05	1.50
$\hat{\sigma_4}$ (ITT_{js})	0.10	0.06	0.03	0.06	0.09	0.13	0.23
$\hat{\sigma_5}$ (ITT_{js})	4.64	0.90	3.11	4.01	4.57	5.19	6.62
$\hat{\sigma_6}$ ($ITT_{js} * LEN_j$)	0.07	0.04	0.02	0.04	0.07	0.10	0.17
**M3**							
$\hat{b_1}$ (FRW_j)	2.11	0.80	0.61	1.56	2.10	2.63	3.75
$\hat{b_2}$ (DIR_j)	4.56	1.87	0.92	3.31	4.58	5.88	8.19
$\hat{b_3}$ (TNR_j)	-0.20	0.30	-0.81	-0.40	-0.20	0.01	0.38
$\hat{b_4}$ (LEN_j)	-0.14	0.16	-0.46	-0.25	-0.14	-0.03	0.17
$\hat{b_5}$ (ITT_{js})	-5.28	1.24	-7.76	-6.10	-5.26	-4.43	-2.87
$\hat{b_6}$ ($ITT_{js} * LEN_j$)	0.13	0.10	-0.06	0.06	0.12	0.20	0.33
$\hat{\sigma_1}$ (FRW_j)	2.33	1.15	0.83	1.39	2.04	3.10	4.91
$\hat{\sigma_2}$ (DIR_j)	3.00	2.66	0.79	1.25	1.95	3.43	10.57
$\hat{\sigma_3}$ (TNR_j)	1.24	0.29	0.76	1.04	1.22	1.43	1.91
$\hat{\sigma_4}$ (ITT_{js})	0.51	0.13	0.37	0.44	0.48	0.55	0.85
$\hat{\sigma_5}$ (ITT_{js})	4.72	0.91	3.11	4.08	4.66	5.29	6.66
$\hat{\sigma_{12}}$ (FRW_j-DIR_j)	1.96	8.73	-14.89	-0.84	0.51	3.68	25.0
$\hat{\sigma_{13}}$ (FRW_j-TNR_j)	1.21	1.60	-0.85	0.15	0.79	1.85	5.48
$\hat{\sigma_{14}}$ (FRW_j-ITT_{js})	-0.36	0.65	-2.21	-0.53	-0.19	-0.02	0.58
$\hat{\sigma_{23}}$ (DIR_j-TNR_j)	0.63	2.28	-2.96	-0.39	0.30	1.22	6.41
$\hat{\sigma_{24}}$ (DIR_j-ITT_{js})	0.83	2.06	-0.35	-0.01	0.15	0.58	7.09
$\hat{\sigma_{34}}$ (TNR_j-ITT_{js})	0.00	0.19	-0.45	-0.10	0.01	0.11	0.38

Mean, standard deviation and some quantiles of the sampled posterior distributions of the parameters of the MXL models.
