# Supplementary material for: Unravelling travellers’ route choice behaviour at full-scale urban network by focusing on representative OD pairs in computer experiments
Source: PLoS One. 2019 Nov 12;14(11):e0225069. doi: 10.1371/journal.pone.0225069 (PMC6850682; doi:10.1371/journal.pone.0225069)
Supplement: S2 Table — (PDF) [file pone.0225069.s004.pdf]

Computational effort in the estimation of the MXL models

Table A: **Execution time (in seconds) to draw 30,000 samples of the posterior distribution of the parameters.**

**Model**	**user**	**system**	**elapsed**
M1	347.983	0.549	350.600
M2	452.597	0.959	458.878
M3	372.546	1.014	376.076

* Estimation for the 9 cluster centroids which are composed of 802 route choices.

Table B: **Hardware and software specifications.**

**Hardware**	
Model Name	iMac
Processor Name	Intel Core i5
Processor Speed	3.2 GHz
Number of Processors	1
Total Number of Cores	4
L2 Cache (per Core)	256 KB
L3 Cache	6 MB
Memory	16 GB

**Software**	
Operating System	macOS 10.14.2 (18C54)
R version	R version 3.5.1 (2018-07-02)
JAGS version	4.3.0
